# Supplementary material for: Genetic Predisposition to SARS-CoV-2 Infection: Cytokine Polymorphism and Disease Transmission within Households
Source: Biology (Basel). 2023 Oct 30;12(11):1385. doi: 10.3390/biology12111385 (PMC10669642; doi:10.3390/biology12111385)
Supplement: Supplementary file 1 [file biology-12-01385-s001.zip › biology-2612320-supplementary.pdf]

Supplement

Genetic Predisposition to SARS-CoV-2 Infection: Cytokine Polymorphism and Disease Transmission within Households

Marius Saal, Henry Loeffler-Wirth, Thomas Gruenewald, Ilias Doxiadis and Claudia Lehmann

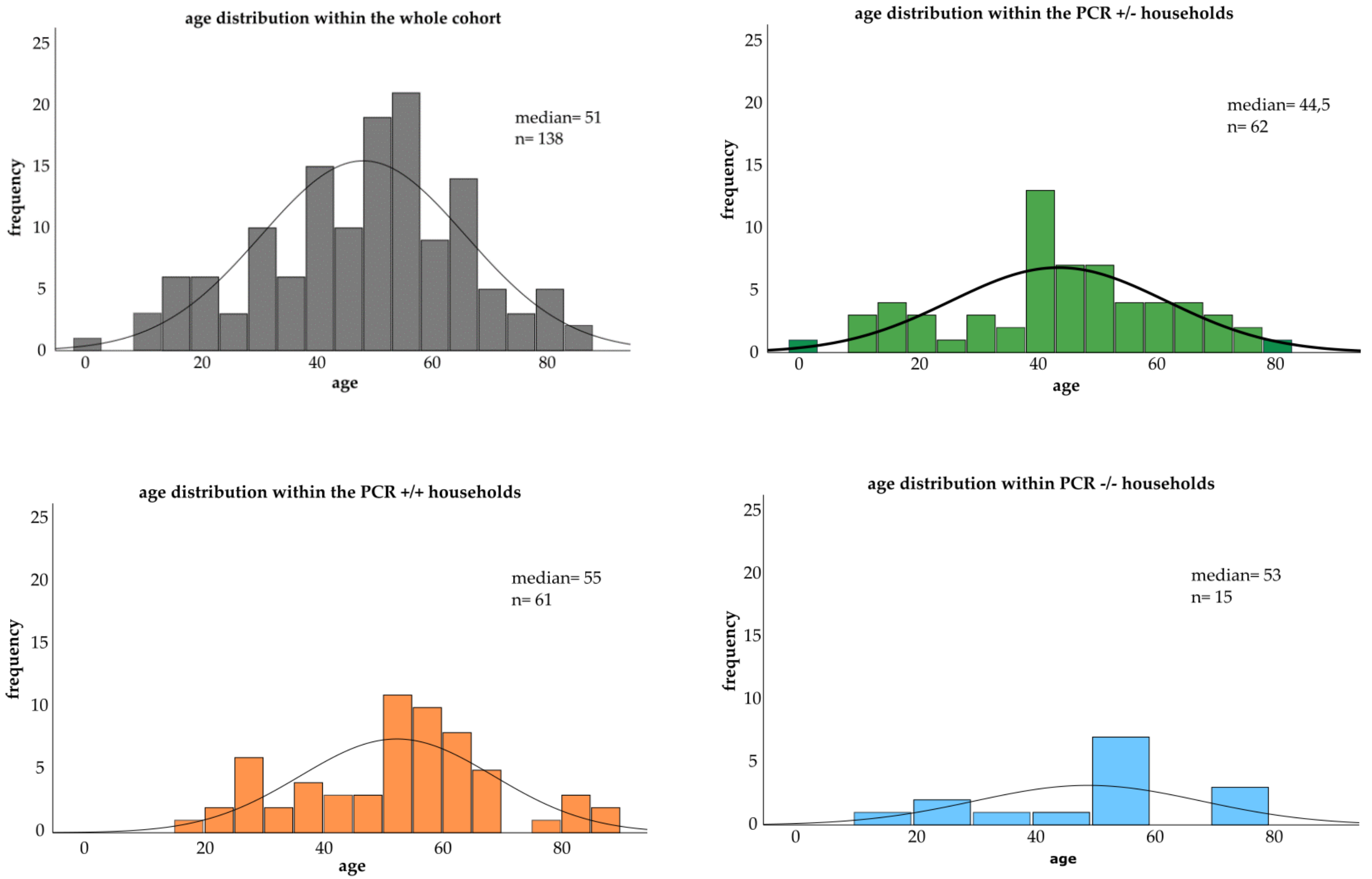

Figure S1. Histogram of age distribution of the different PCR household groups.

Table S1. Cytokine polymorphism in gene expression levels in various populations. *Source: www.allelefrequencies.net/default.asp*

**Cytokine region**	**Polymor-phism**	**Gene expression level**	**Saxon population%**	**Germany %**	**Brazil %**	**England %**	**Greece%**	**Portugal Center %**	**USA%**	**Japan%**	**South Africa%**	**Zimbabwe%**
TNF-α promoter -308G	G/G	low	76	70.7-73.0	71.0-80.0	60.5-76.8	68.0-86.0	65.0	65.7-81.0	96.7	62.8	na
TNF-α promoter -308A -308G	G/A	high	23	24.3-28.5	18.0-27.0	20.8-35.5	14.0-32.0	21.0	15.2-31.6	3.1	30.2	na
TNF-α promoter -308A	A/A	high	2	0.5-2.7	2.0	2.4-16.0	0.0	14.0	1.1-3.8	0.2	7.0	na
IFN-γ intron1 +874T	T/T	high	20	na	5.0-18.0	17.9-21.0	25.6	18.8 Azores	0.0-25.4	na	na	na
IFN-γ intron1 +874T +874A	T/A	intermediate	51	na	48.0-54.0	48.2-56.7	47.4	50.4 Azores	26.7.-57.2	na	na	na
IFN-γ intron1 +874A	A/A	low	30	na	31.0-47.0	25.0-31.1	26.9	30.8 Azores	21.9-66.7	na	na	na
IL-6 promoter -174G	G/G	high	30	31.0-38.7	49.3-72.0	30.2-43.4	51.3-65.0	43.0	26.7-93.3	na	98.8	100.0
IL-6 promoter -174C -174G	G/C	high	53	44.0-48.0	26.0-43.8	45.1-52.5	31.0-35.0	39.0	3.0-52.0	na	1.2	0.0
IL-6 promoter -174C	C/C	low	17	17.1-21.0	2.0-12.0	15.2-19.6	0.0-14.1	18.0	0.0-37.6	na	0.0	0.0
IL-10 promoter -1082A	A/A	low	27	24.5-35.4	39.0-44.4.	20.6-28.0	37.0-42.0	36.0	16.0-51.4	na	33.4	na
IL-10 promoter -1082G	G/G	high	24	17.3-28.8	10.7-19.0	25.6-49.9	10.0-20.5	12.0	4.3-32.0	na	9.5	na
IL-10 promoter -1082A -1082G	G/A	intermediate	48	41.4-58.0	40.0-49.6	27.7-53.8	42.3-53.0	52.0	39.8-60.9	na	57.1	na
IL-10 promoter -819C	C/C	high/intermediate/low	58	57.8-59.0	37.0-49.0	57.6-69.9	33.0-55.1	51.0	31.6-69.0	na	38.0	na
IL-10 promoter -819T	T/T	low	9	5.0-6.3	7.0-17.0	2.7-8.0	9.0-17.0	10.0	6.0-25.0	na	6.0	na
IL-10 promoter -819T -819C	C/T	intermediate/low	34	35.1-36.4	37.0-48.0	27.4-37.3	34.6-51.0	39.0	25.0-57.9	na	56.0	na
IL-10 promoter -592A	A/A	low	9	5.0	7.0-17.0	2.7-8.0	8.0-17.0	10.0	6.0-25.0	na	6.0	na
IL-10 promoter -592C	C/C	high/intermediate/low	58	59.0	37.0-49.0	60.2-69.9	35.0-55.1	51.0	31.6-69.0	na	38.0	na
IL-10 promoter -592A -592C	C/A	intermediate/low	34	36.0	37.0-48.0	27.4-37.3	34.6-49.0	39.0	25.0-57.9	na	56.0	na
TGF-β1 codon 10T	T/T	**high**/ intermediate (0)/low (0)	41	34.5	27.0-36.7	41.0-42.0	22.0-28.0	28.0	28.0-44.0	na	na	na
TGF-β1 codon 10C	C/C	**intermediate (7)/low (3,1)**	10	13.0	16.6-25.0	11.0-14.0	3.0-22.0	25.0	7.8-21.1	na	na	na
TGF-β1 codon 10C codon 10T	T/C	**high (35,9)/intermediate (12,5)/** low	48	52.5	45.9-53.1	44.0-48.0	50.0-75.0	47.0	42.1-57.8	na	na	na
TGF-β1 codon 25C	C/C	low	0.0	0.0	0.0-2.0	0.6-2.0	0.0	1.0	0.0-5.3	na	na	na
TGF-β1 codon 25G	G/G	**high (41,4)/intermediate (42,5)**	84	85.0	85.0-89.0	81.0-83.1	88.0-90.0	84.0	80.0-93.0	na	na	na
TGF-β1 codon 25G codon 25C	G/C	**intermediate (12,5)/low (3,1)**	16	15.0	10.0-13.6	15.0-18.0	10.0-12.0	15.0	7.0-20.0	na	na	na

Table S2. Inheritance of the cytokine polymorphism in families.

household number	genetic relation	PCR houshold	SARS-CoV-2 (1=PCR pos; 4=PCR neg)	TNF-α	cytokine release potential TNF-α	TGF-β1	cytokine release potential TGF-β1	IL-10	cytokine release potential IL-10	IL-6	cytokine release potential IL-6	IFN-γ	cytokine release potential IFN-γ
LEI_001	M	+/- PCR	1	G/G	low	T/T G/G	high	ACC/ACC	low	G/C	high	T/A	intermediate
LEI_001	F2	+/- PCR	1	G/G	low	T/T G/G	high	GCC/GCC	high	G/C	high	T/T	high
LEI_001	C1F2M	+/- PCR	1	G/G	low	T/T G/G	high	GCC/ACC	intermediate	G/G	high	T/T	high
LEI_001	C2	+/- PCR	1	G/G	low	T/C G/G	high	ACC/ACC	low	G/C	high	T/A	intermediate
LEI_001	C1	+/- PCR	4	G/G	low	T/C G/G	high	GCC/ACC	intermediate	G/C	high	T/A	intermediate
LEI_007	MM	-/- PCR	4	G/G	low	T/C G/C	intermediate	GCC/GCC	high	G/G	high	T/A*	intermediate
LEI_007	FM	-/- PCR	4	G/G	low	T/T G/G	high	GCC/ACC	intermediate	G/G	high	T/A*	intermediate
LEI_007	C1	-/- PCR	4	G/G	low	T/C G/C	intermediate	GCC/GCC	high	G/G	high	T/A	intermediate
LEI_007	C2	-/- PCR	4	G/G	low	T/C G/C	intermediate	GCC/GCC	high	G/G	high	T/A*	intermediate
LEI_007	F	-/- PCR	4	G/G	low	T/T G/G	high	ACC/ATA	low	G/C	high	T/A	intermediate
LEI_007	C1C1	-/- PCR	4	G/G	low	T/C G/C	intermediate	GCC/ATA	intermediate	G/G	high	A/A	low
LEI_007	C2C1	-/- PCR	4	G/G	low	T/C G/C	intermediate	GCC/ATA	intermediate	G/G	high	A/A	low
LEI_008	M	-/- PCR	4	G/G	low	T/C G/G	high	GCC/ACC	intermediate	G/G	high	T/A	intermediate
LEI_008	F	-/- PCR	4	G/G	low	C/C G/G	intermediate	GCC/ATA	intermediate	G/C	high	T/T	high
LEI_008	C1	-/- PCR	4	G/G	low	C/C G/G	intermediate	GCC/ATA	intermediate	G/G	high	T/T	high
LEI_033	M	+/+ PCR	1	G/G	low	T/T G/G	high	GCC/ACC	intermediate	G/C*	high	T/A	intermediate
LEI_033	F	+/+ PCR	1	G/G	low	T/T G/G	high	GCC/ACC	intermediate	G/C*	high	A/A	low
LEI_033	C1	+/+ PCR	1	G/G	low	T/T G/G	high	GCC/GCC	high	G/C*	high	T/A	intermediate
LEI_039	M	+/+ PCR	1	G/G	low	T/C G/G	high	ACC/ATA	low	G/C	high	A/A	low
LEI_039	F	+/+ PCR	1	G/G	low	T/C G/C	intermediate	ATA/ATA	low	G/C	high	T/A	intermediate
LEI_039	C1	+/+ PCR	1	G/G	low	T/C G/G	high	ATA/ATA	low	G/G	high	A/A	low
LEI_041	MM	+/- PCR	1	G/G	low	T/C G/G	high	ATA/ATA	low	G/C*	high	T/A	intermediate
LEI_041	FM	+/- PCR	4	G/G	low	T/C G/G	high	GCC/ATA	intermediate	G/C*	high	T/T	high
LEI_041	C1	+/- PCR	1	G/G	low	T/T G/G	high	GCC/ATA	intermediate	G/C*	high	T/T	high
LEI_041	F	+/- PCR	1	G/G	low	T/C G/G	high	GCC/ACC	intermediate	G/G	high	T/T	high
LEI_041	C1C1	+/- PCR	4	G/G	low	T/C G/G	high	ACC/ATA	low	G/C	high	T/T	high
LEI_041	C2C1	+/- PCR	1	G/G	low	T/C G/G	high	ACC/ATA	low	G/G	high	T/T	high
LEI_043	M	+/- PCR	4	G/G	low	T/T G/G	high	GCC/ATA	intermediate	G/C	high	A/A	low
LEI_043	F	+/- PCR	1	G/G	low	T/C G/C	intermediate	GCC/ACC	intermediate	G/G	high	T/T	high
LEI_043	C1	+/- PCR	4	G/G	low	T/C G/C	intermediate	ACC/ATA	low	G/C	high	T/A	intermediate

[1] Blue colour shows the genotype inherited from the mother (M) and yellow the genotype not inherited from the mother. Red and green colour indicate the genotype inherited from the father (F) and black and pink colour the genotype not inherited from the father. Green C1 and C2: children of M and F, MM: mother of the mother; FM: Father of the mother, C1C1: first child from C1, C2C1: second child from C1. F2: father, who is not genetically related to all children, C1F2M: first child of F2 and M. * marks polymorphisms in which the inheritance from mother or father to the children cannot be clearly stated.
